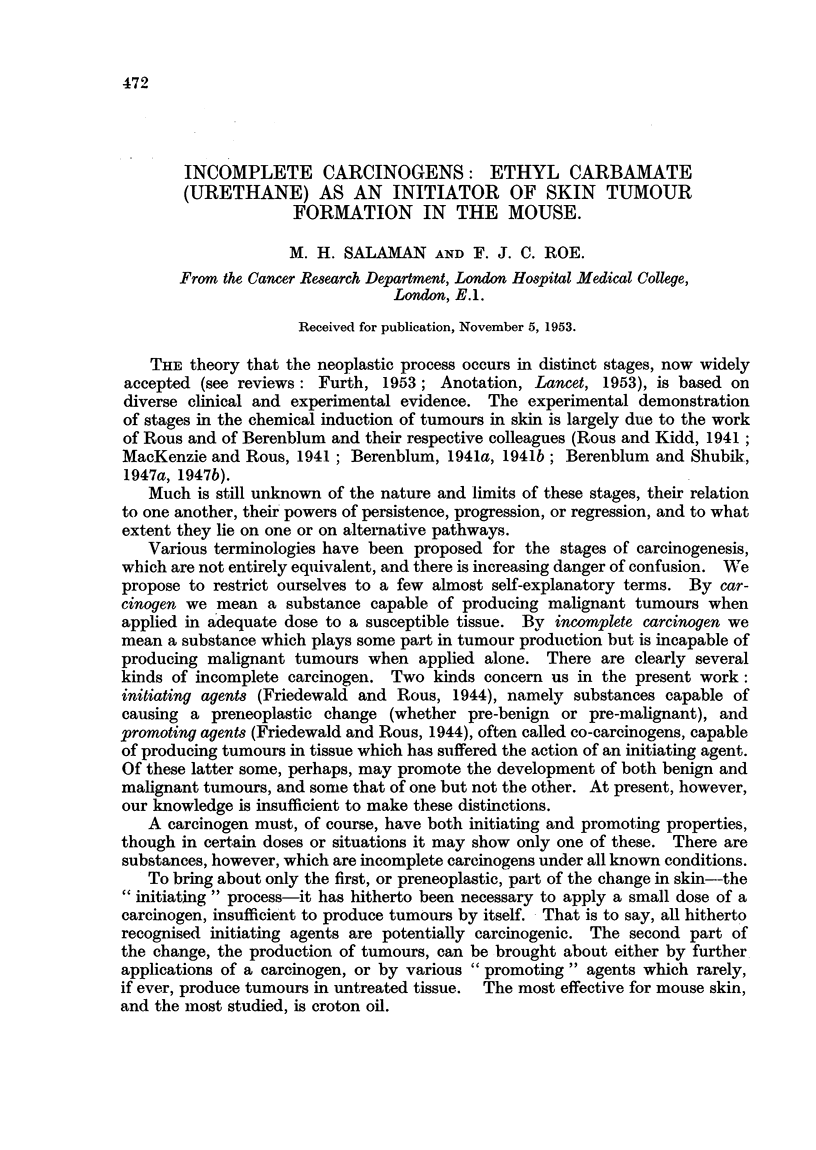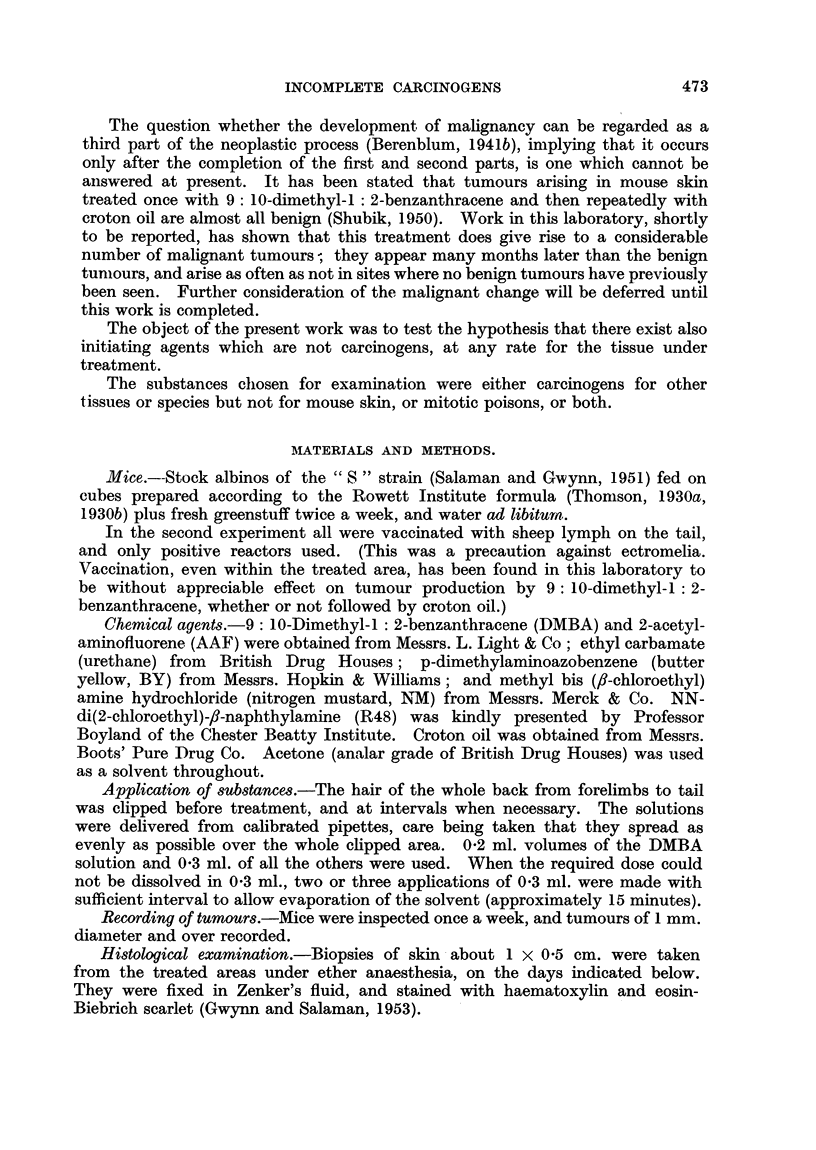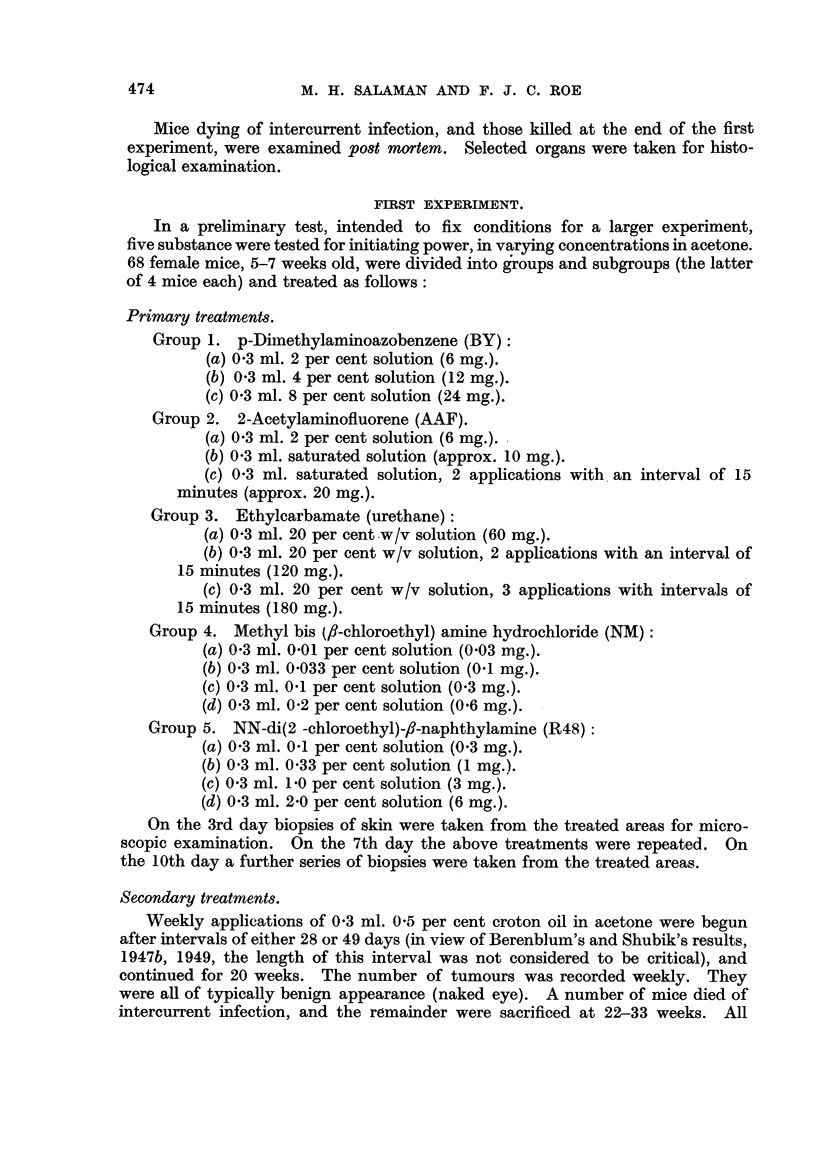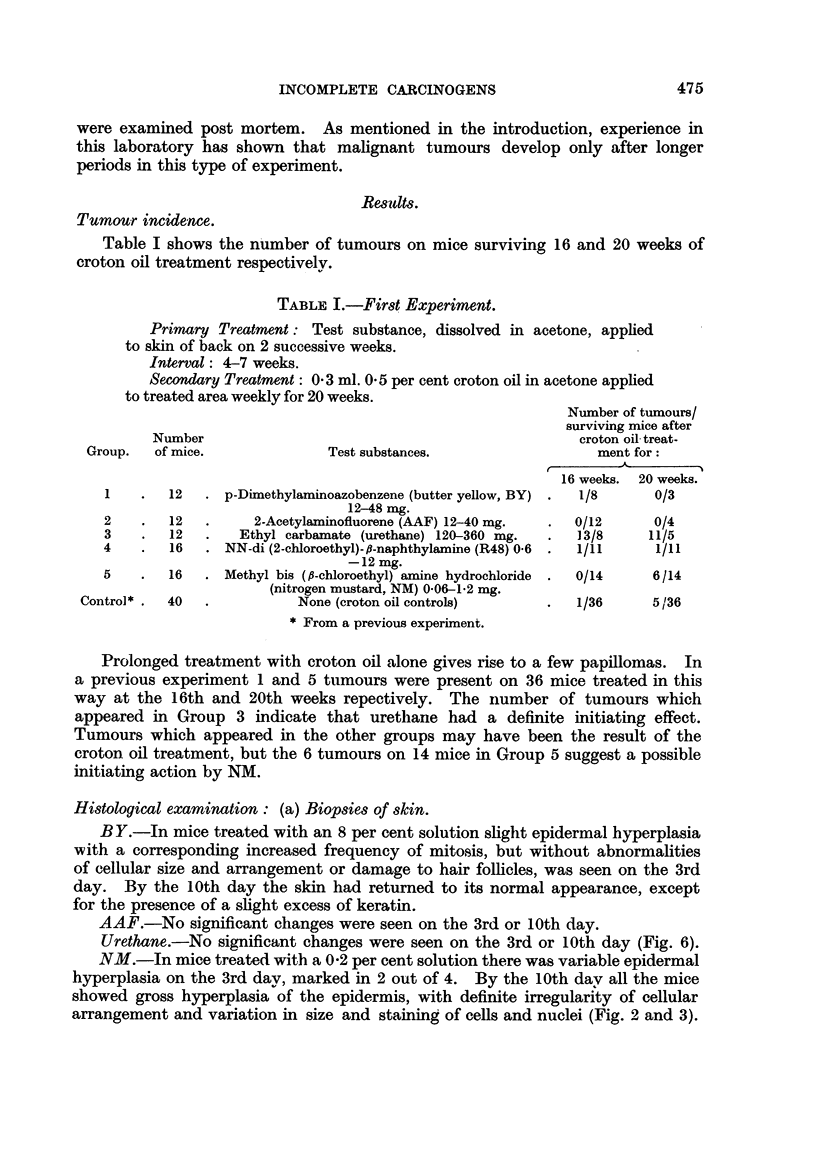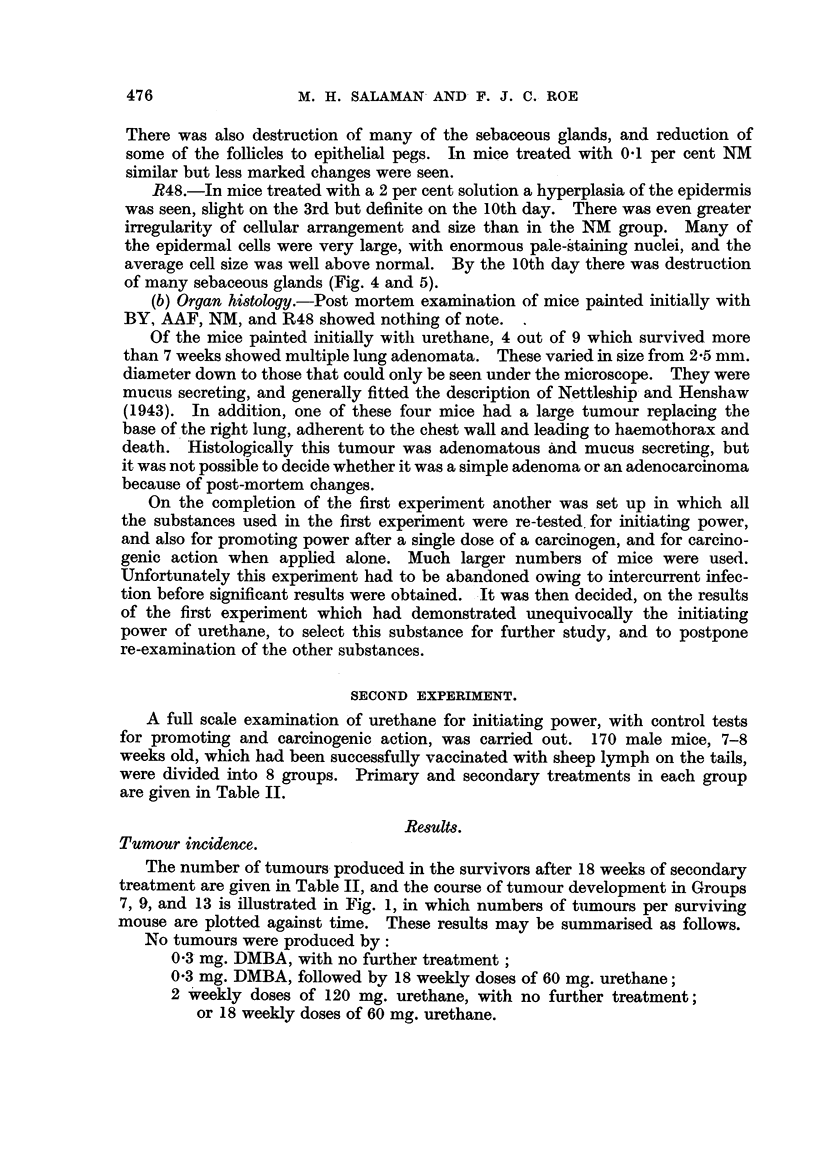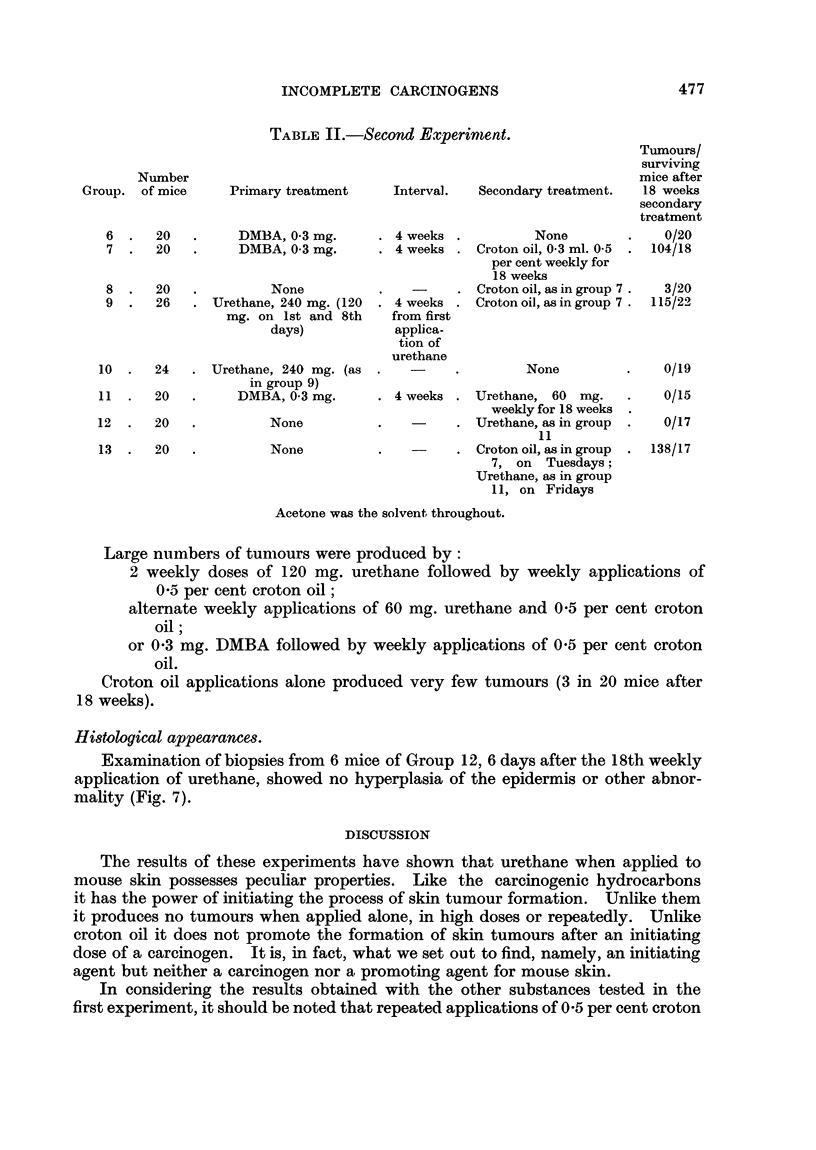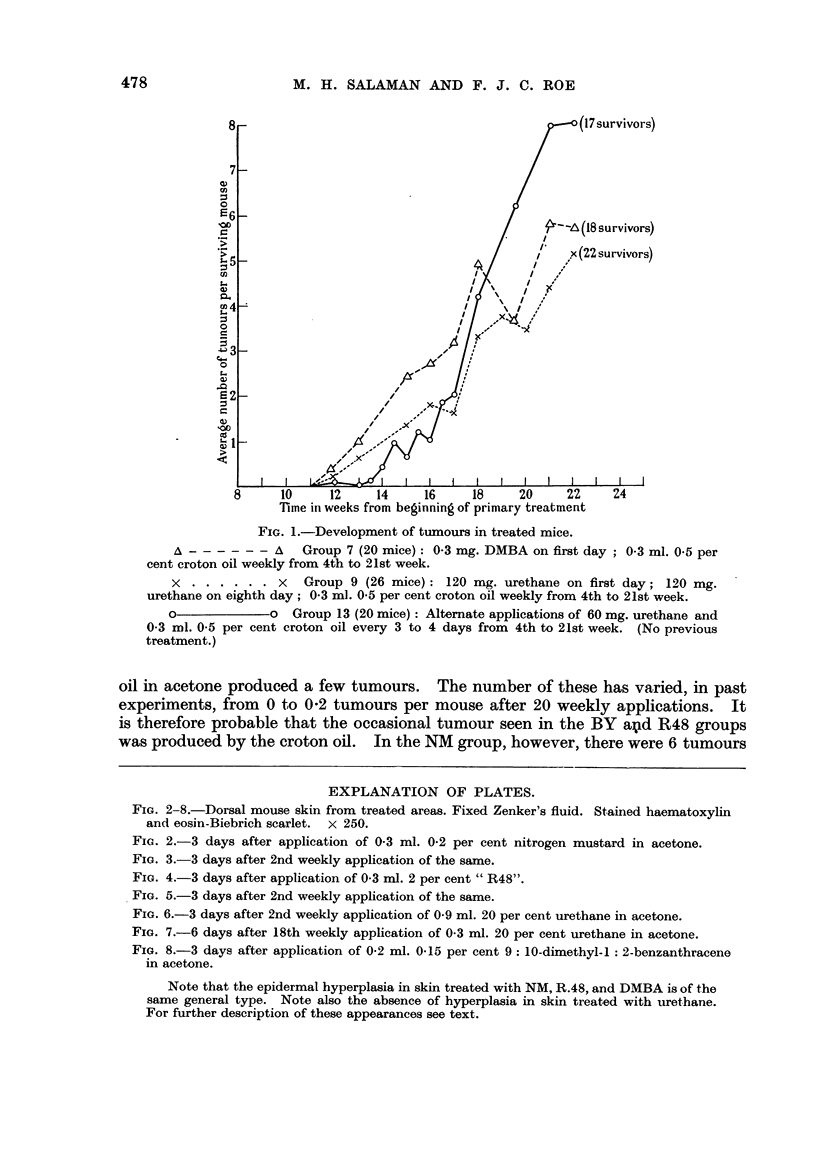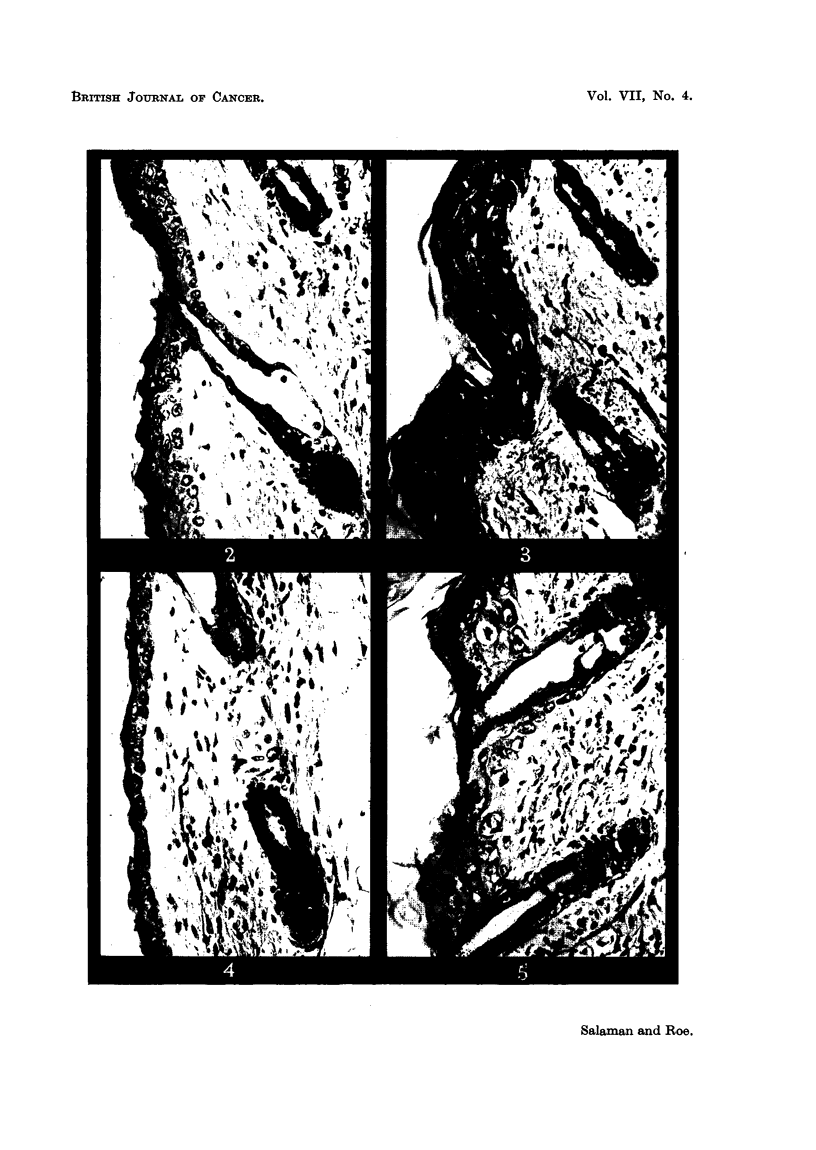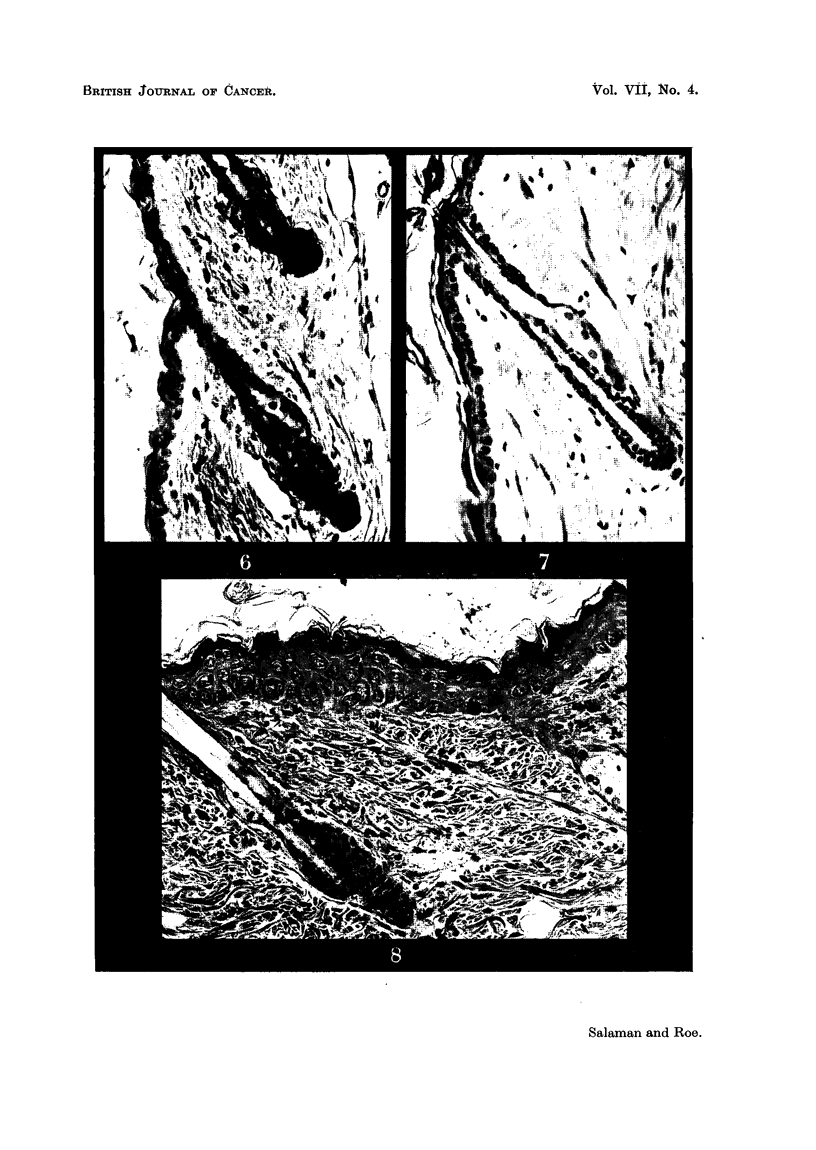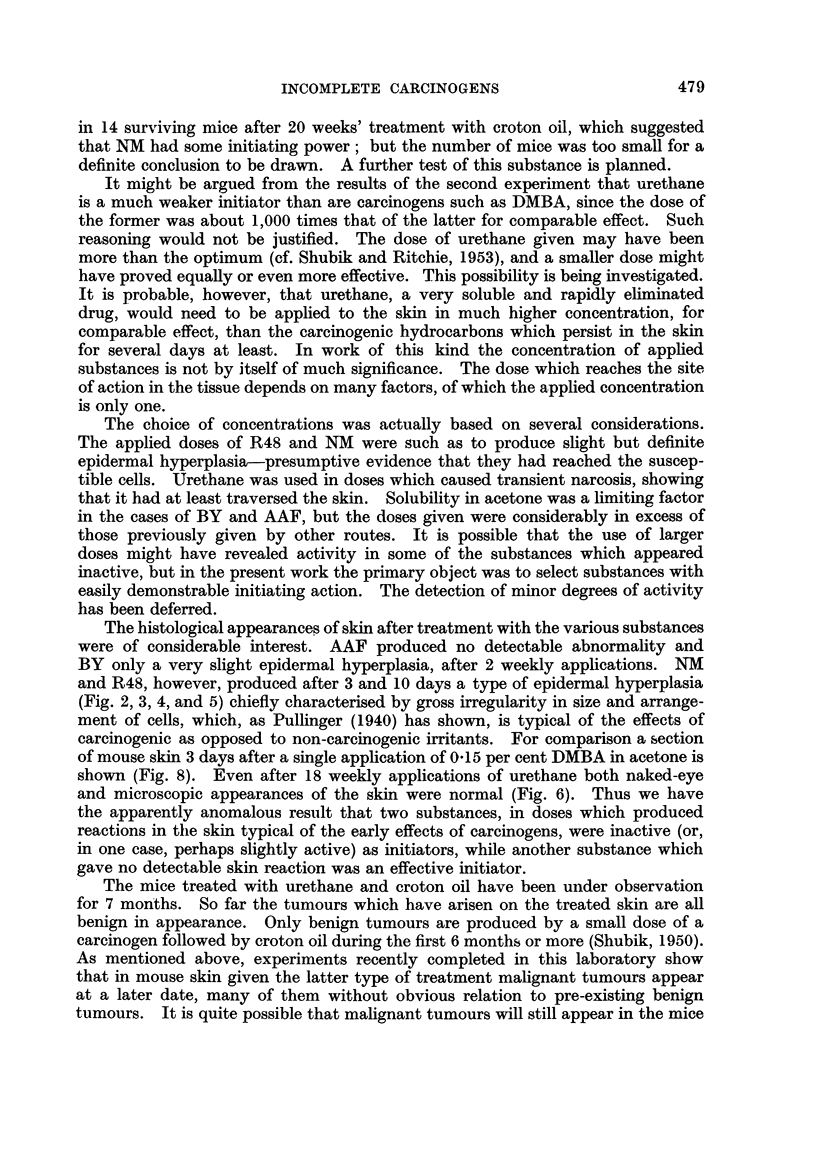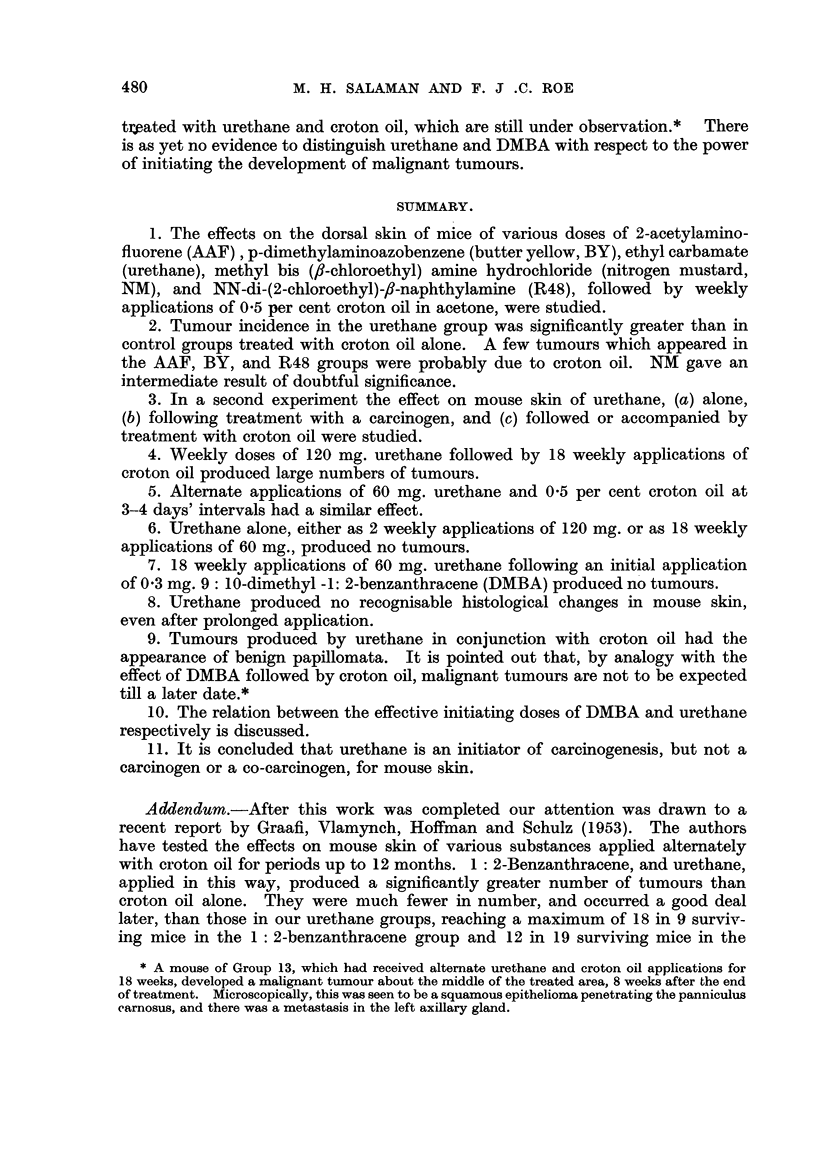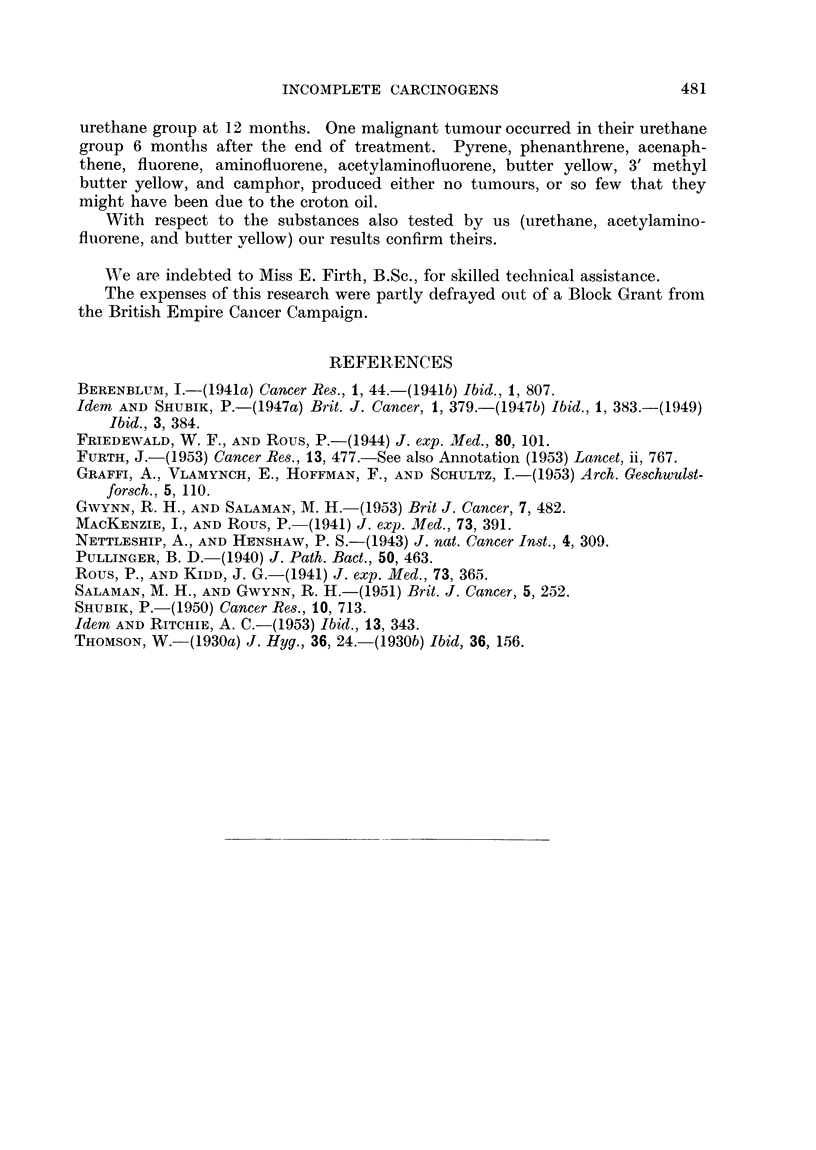# Incomplete Carcinogens: Ethyl Carbamate (Urethane) as an Initiator of Skin Tumour Formation in the Mouse

**DOI:** 10.1038/bjc.1953.49

**Published:** 1953-12

**Authors:** M. H. Salaman, F. J. C. Roe

## Abstract

**Images:**


### INCOMPLETE CARCINOGENS: ETHYL CARBAMATE (URETHANE) AS AN INITIATOR OF SKIN TUMOUR FORMATION IN THE MOUSE.

M. H. SALAMAN AND F. J. C. ROE.

*From the Cancer Research Department, London Hospital Medical College, London, E.1.*

Received for publication, November 5, 1953.

THE theory that the neoplastic process occurs in distinct stages, now widely accepted (see reviews: Furth, 1953; Anotation, *Lancet*, 1953), is based on diverse clinical and experimental evidence. The experimental demonstration of stages in the chemical induction of tumours in skin is largely due to the work of Rous and of Berenblum and their respective colleagues (Rous and Kidd, 1941; MacKenzie and Rous, 1941; Berenblum, 1941*a*, 1941*b*; Berenblum and Shubik, 1947*a*, 1947*b*).

Much is still unknown of the nature and limits of these stages, their relation to one another, their powers of persistence, progression, or regression, and to what extent they lie on one or on alternative pathways.

Various terminologies have been proposed for the stages of carcinogenesis, which are not entirely equivalent, and there is increasing danger of confusion. We propose to restrict ourselves to a few almost self-explanatory terms. By *carcinogen* we mean a substance capable of producing malignant tumours when applied in adequate dose to a susceptible tissue. By *incomplete carcinogen* we mean a substance which plays some part in tumour production but is incapable of producing malignant tumours when applied alone. There are clearly several kinds of incomplete carcinogen. Two kinds concern us in the present work: *initiating agents* (Friedewald and Rous, 1944), namely substances capable of causing a preneoplastic change (whether pre-benign or pre-malignant), and *promoting agents* (Friedewald and Rous, 1944), often called co-carcinogens, capable of producing tumours in tissue which has suffered the action of an initiating agent. Of these latter some, perhaps, may promote the development of both benign and malignant tumours, and some that of one but not the other. At present, however, our knowledge is insufficient to make these distinctions.

A carcinogen must, of course, have both initiating and promoting properties, though in certain doses or situations it may show only one of these. There are substances, however, which are incomplete carcinogens under all known conditions.

To bring about only the first, or preneoplastic, part of the change in skin—the "initiating" process—it has hitherto been necessary to apply a small dose of a carcinogen, insufficient to produce tumours by itself. That is to say, all hitherto recognised initiating agents are potentially carcinogenic. The second part of the change, the production of tumours, can be brought about either by further applications of a carcinogen, or by various "promoting" agents which rarely, if ever, produce tumours in untreated tissue. The most effective for mouse skin, and the most studied, is croton oil.

The question whether the development of malignancy can be regarded as a third part of the neoplastic process (Berenblum, 1941*b*), implying that it occurs only after the completion of the first and second parts, is one which cannot be answered at present. It has been stated that tumours arising in mouse skin treated once with 9 : 10-dimethyl-1 : 2-benzanthracene and then repeatedly with croton oil are almost all benign (Shubik, 1950). Work in this laboratory, shortly to be reported, has shown that this treatment does give rise to a considerable number of malignant tumours ; they appear many months later than the benign tumours, and arise as often as not in sites where no benign tumours have previously been seen. Further consideration of the malignant change will be deferred until this work is completed.

The object of the present work was to test the hypothesis that there exist also initiating agents which are not carcinogens, at any rate for the tissue under treatment.

The substances chosen for examination were either carcinogens for other tissues or species but not for mouse skin, or mitotic poisons, or both.

### MATERIALS AND METHODS.

*Mice.*—Stock albinos of the " S " strain (Salaman and Gwynn, 1951) fed on cubes prepared according to the Rowett Institute formula (Thomson, 1930*a*, 1930*b*) plus fresh greenstuff twice a week, and water *ad libitum*.

In the second experiment all were vaccinated with sheep lymph on the tail, and only positive reactors used. (This was a precaution against ectromelia. Vaccination, even within the treated area, has been found in this laboratory to be without appreciable effect on tumour production by 9 : 10-dimethyl-1 : 2-benzanthracene, whether or not followed by croton oil.)

*Chemical agents.*—9 : 10-Dimethyl-1 : 2-benzanthracene (DMBA) and 2-acetyl-aminofluorene (AAF) were obtained from Messrs. L. Light & Co ; ethyl carbamate (urethane) from British Drug Houses ; p-dimethylaminoazobenzene (butter yellow, BY) from Messrs. Hopkin & Williams ; and methyl bis (β-chloroethyl) amine hydrochloride (nitrogen mustard, NM) from Messrs. Merck & Co. NN-di(2-chloroethyl)-β-naphthylamine (R48) was kindly presented by Professor Boyland of the Chester Beatty Institute. Croton oil was obtained from Messrs. Boots' Pure Drug Co. Acetone (analar grade of British Drug Houses) was used as a solvent throughout.

*Application of substances.*—The hair of the whole back from forelimbs to tail was clipped before treatment, and at intervals when necessary. The solutions were delivered from calibrated pipettes, care being taken that they spread as evenly as possible over the whole clipped area. 0·2 ml. volumes of the DMBA solution and 0·3 ml. of all the others were used. When the required dose could not be dissolved in 0·3 ml., two or three applications of 0·3 ml. were made with sufficient interval to allow evaporation of the solvent (approximately 15 minutes).

*Recording of tumours.*—Mice were inspected once a week, and tumours of 1 mm. diameter and over recorded.

*Histological examination.*—Biopsies of skin about 1 × 0·5 cm. were taken from the treated areas under ether anaesthesia, on the days indicated below. They were fixed in Zenker's fluid, and stained with haematoxylin and eosin-Biebrich scarlet (Gwynn and Salaman, 1953).

Mice dying of intercurrent infection, and those killed at the end of the first experiment, were examined *post mortem*. Selected organs were taken for histological examination.

FIRST EXPERIMENT.

In a preliminary test, intended to fix conditions for a larger experiment, five substance were tested for initiating power, in varying concentrations in acetone. 68 female mice, 5–7 weeks old, were divided into groups and subgroups (the latter of 4 mice each) and treated as follows :

*Primary treatments.*

Group 1. p-Dimethylaminoazobenzene (BY) :
(*a*) 0·3 ml. 2 per cent solution (6 mg.).
(*b*) 0·3 ml. 4 per cent solution (12 mg.).
(*c*) 0·3 ml. 8 per cent solution (24 mg.).

Group 2. 2-Acetylaminofluorene (AAF).
(*a*) 0·3 ml. 2 per cent solution (6 mg.).
(*b*) 0·3 ml. saturated solution (approx. 10 mg.).
(*c*) 0·3 ml. saturated solution, 2 applications with an interval of 15 minutes (approx. 20 mg.).

Group 3. Ethylcarbamate (urethane) :
(*a*) 0·3 ml. 20 per cent w/v solution (60 mg.).
(*b*) 0·3 ml. 20 per cent w/v solution, 2 applications with an interval of 15 minutes (120 mg.).
(*c*) 0·3 ml. 20 per cent w/v solution, 3 applications with intervals of 15 minutes (180 mg.).

Group 4. Methyl bis ($\beta$-chloroethyl) amine hydrochloride (NM) :
(*a*) 0·3 ml. 0·01 per cent solution (0·03 mg.).
(*b*) 0·3 ml. 0·033 per cent solution (0·1 mg.).
(*c*) 0·3 ml. 0·1 per cent solution (0·3 mg.).
(*d*) 0·3 ml. 0·2 per cent solution (0·6 mg.).

Group 5. NN-di(2 -chloroethyl)-$\beta$-naphthylamine (R48) :
(*a*) 0·3 ml. 0·1 per cent solution (0·3 mg.).
(*b*) 0·3 ml. 0·33 per cent solution (1 mg.).
(*c*) 0·3 ml. 1·0 per cent solution (3 mg.).
(*d*) 0·3 ml. 2·0 per cent solution (6 mg.).

On the 3rd day biopsies of skin were taken from the treated areas for microscopic examination. On the 7th day the above treatments were repeated. On the 10th day a further series of biopsies were taken from the treated areas.

*Secondary treatments.*

Weekly applications of 0·3 ml. 0·5 per cent croton oil in acetone were begun after intervals of either 28 or 49 days (in view of Berenblum's and Shubik's results, 1947*b*, 1949, the length of this interval was not considered to be critical), and continued for 20 weeks. The number of tumours was recorded weekly. They were all of typically benign appearance (naked eye). A number of mice died of intercurrent infection, and the remainder were sacrificed at 22–33 weeks. All were examined post mortem. As mentioned in the introduction, experience in this laboratory has shown that malignant tumours develop only after longer periods in this type of experiment.

### *Results.*

### *Tumour incidence.*

Table I shows the number of tumours on mice surviving 16 and 20 weeks of croton oil treatment respectively.

TABLE I.—*First Experiment.*

*Primary Treatment:* Test substance, dissolved in acetone, applied to skin of back on 2 successive weeks.

*Interval*: 4–7 weeks.

*Secondary Treatment*: 0·3 ml. 0·5 per cent croton oil in acetone applied to treated area weekly for 20 weeks.

| Group. | Number of mice. | Test substances. | Number of tumours/surviving mice after croton oil treatment for: 16 weeks. | 20 weeks. |
|---|---|---|---|---|
| 1 | 12 | p-Dimethylaminoazobenzene (butter yellow, BY) 12–48 mg. | 1/8 | 0/3 |
| 2 | 12 | 2-Acetylaminofluorene (AAF) 12–40 mg. | 0/12 | 0/4 |
| 3 | 12 | Ethyl carbamate (urethane) 120–360 mg. | 13/8 | 11/5 |
| 4 | 16 | NN-di (2-chloroethyl)-β-naphthylamine (R48) 0·6 —12 mg. | 1/11 | 1/11 |
| 5 | 16 | Methyl bis (β-chloroethyl) amine hydrochloride (nitrogen mustard, NM) 0·06–1·2 mg. | 0/14 | 6/14 |
| Control* | 40 | None (croton oil controls) | 1/36 | 5/36 |

* From a previous experiment.

Prolonged treatment with croton oil alone gives rise to a few papillomas. In a previous experiment 1 and 5 tumours were present on 36 mice treated in this way at the 16th and 20th weeks repectively. The number of tumours which appeared in Group 3 indicate that urethane had a definite initiating effect. Tumours which appeared in the other groups may have been the result of the croton oil treatment, but the 6 tumours on 14 mice in Group 5 suggest a possible initiating action by NM.

*Histological examination :* (a) *Biopsies of skin.*

*BY.*—In mice treated with an 8 per cent solution slight epidermal hyperplasia with a corresponding increased frequency of mitosis, but without abnormalities of cellular size and arrangement or damage to hair follicles, was seen on the 3rd day. By the 10th day the skin had returned to its normal appearance, except for the presence of a slight excess of keratin.

*AAF.*—No significant changes were seen on the 3rd or 10th day.

*Urethane.*—No significant changes were seen on the 3rd or 10th day (Fig. 6).

*NM.*—In mice treated with a 0·2 per cent solution there was variable epidermal hyperplasia on the 3rd day, marked in 2 out of 4. By the 10th day all the mice showed gross hyperplasia of the epidermis, with definite irregularity of cellular arrangement and variation in size and staining of cells and nuclei (Fig. 2 and 3).

There was also destruction of many of the sebaceous glands, and reduction of some of the follicles to epithelial pegs. In mice treated with 0·1 per cent NM similar but less marked changes were seen.

*R*48.—In mice treated with a 2 per cent solution a hyperplasia of the epidermis was seen, slight on the 3rd but definite on the 10th day. There was even greater irregularity of cellular arrangement and size than in the NM group. Many of the epidermal cells were very large, with enormous pale-staining nuclei, and the average cell size was well above normal. By the 10th day there was destruction of many sebaceous glands (Fig. 4 and 5).

(*b*) *Organ histology*.—Post mortem examination of mice painted initially with BY, AAF, NM, and R48 showed nothing of note.

Of the mice painted initially with urethane, 4 out of 9 which survived more than 7 weeks showed multiple lung adenomata. These varied in size from 2·5 mm. diameter down to those that could only be seen under the microscope. They were mucus secreting, and generally fitted the description of Nettleship and Henshaw (1943). In addition, one of these four mice had a large tumour replacing the base of the right lung, adherent to the chest wall and leading to haemothorax and death. Histologically this tumour was adenomatous and mucus secreting, but it was not possible to decide whether it was a simple adenoma or an adenocarcinoma because of post-mortem changes.

On the completion of the first experiment another was set up in which all the substances used in the first experiment were re-tested for initiating power, and also for promoting power after a single dose of a carcinogen, and for carcinogenic action when applied alone. Much larger numbers of mice were used. Unfortunately this experiment had to be abandoned owing to intercurrent infection before significant results were obtained. It was then decided, on the results of the first experiment which had demonstrated unequivocally the initiating power of urethane, to select this substance for further study, and to postpone re-examination of the other substances.

### SECOND EXPERIMENT.

A full scale examination of urethane for initiating power, with control tests for promoting and carcinogenic action, was carried out. 170 male mice, 7–8 weeks old, which had been successfully vaccinated with sheep lymph on the tails, were divided into 8 groups. Primary and secondary treatments in each group are given in Table II.

### *Results.*

*Tumour incidence.*

The number of tumours produced in the survivors after 18 weeks of secondary treatment are given in Table II, and the course of tumour development in Groups 7, 9, and 13 is illustrated in Fig. 1, in which numbers of tumours per surviving mouse are plotted against time. These results may be summarised as follows.

No tumours were produced by :

0·3 mg. DMBA, with no further treatment ;

0·3 mg. DMBA, followed by 18 weekly doses of 60 mg. urethane ;

2 weekly doses of 120 mg. urethane, with no further treatment ;
or 18 weekly doses of 60 mg. urethane.

TABLE II.—*Second Experiment.*

| Group. | Number of mice | Primary treatment | Interval. | Secondary treatment. | Tumours/ surviving mice after 18 weeks secondary treatment |
|---|---|---|---|---|---|
| 6 | 20 | DMBA, 0·3 mg. | 4 weeks | None | 0/20 |
| 7 | 20 | DMBA, 0·3 mg. | 4 weeks | Croton oil, 0·3 ml. 0·5 per cent weekly for 18 weeks | 104/18 |
| 8 | 20 | None | — | Croton oil, as in group 7 | 3/20 |
| 9 | 26 | Urethane, 240 mg. (120 mg. on 1st and 8th days) | 4 weeks from first application of urethane | Croton oil, as in group 7 | 115/22 |
| 10 | 24 | Urethane, 240 mg. (as in group 9) | — | None | 0/19 |
| 11 | 20 | DMBA, 0·3 mg. | 4 weeks | Urethane, 60 mg. weekly for 18 weeks | 0/15 |
| 12 | 20 | None | — | Urethane, as in group 11 | 0/17 |
| 13 | 20 | None | — | Croton oil, as in group 7, on Tuesdays; Urethane, as in group 11, on Fridays | 138/17 |

Acetone was the solvent throughout.

Large numbers of tumours were produced by :

2 weekly doses of 120 mg. urethane followed by weekly applications of 0·5 per cent croton oil; alternate weekly applications of 60 mg. urethane and 0·5 per cent croton oil; or 0·3 mg. DMBA followed by weekly applications of 0·5 per cent croton oil.

Croton oil applications alone produced very few tumours (3 in 20 mice after 18 weeks).

*Histological appearances.*

Examination of biopsies from 6 mice of Group 12, 6 days after the 18th weekly application of urethane, showed no hyperplasia of the epidermis or other abnormality (Fig. 7).

### DISCUSSION

The results of these experiments have shown that urethane when applied to mouse skin possesses peculiar properties. Like the carcinogenic hydrocarbons it has the power of initiating the process of skin tumour formation. Unlike them it produces no tumours when applied alone, in high doses or repeatedly. Unlike croton oil it does not promote the formation of skin tumours after an initiating dose of a carcinogen. It is, in fact, what we set out to find, namely, an initiating agent but neither a carcinogen nor a promoting agent for mouse skin.

In considering the results obtained with the other substances tested in the first experiment, it should be noted that repeated applications of 0·5 per cent croton

FIG. 1.—Development of tumours in treated mice.

△ – – – – – – – △ Group 7 (20 mice) : 0·3 mg. DMBA on first day ; 0·3 ml. 0·5 per cent croton oil weekly from 4th to 21st week.

× . . . . . . × Group 9 (26 mice) : 120 mg. urethane on first day ; 120 mg. urethane on eighth day ; 0·3 ml. 0·5 per cent croton oil weekly from 4th to 21st week.

o————————o Group 13 (20 mice) : Alternate applications of 60 mg. urethane and 0·3 ml. 0·5 per cent croton oil every 3 to 4 days from 4th to 21st week. (No previous treatment.)

oil in acetone produced a few tumours. The number of these has varied, in past experiments, from 0 to 0·2 tumours per mouse after 20 weekly applications. It is therefore probable that the occasional tumour seen in the BY and R48 groups was produced by the croton oil. In the NM group, however, there were 6 tumours

---

EXPLANATION OF PLATES.

FIG. 2–8.—Dorsal mouse skin from treated areas. Fixed Zenker's fluid. Stained haematoxylin and eosin-Biebrich scarlet. × 250.

FIG. 2.—3 days after application of 0·3 ml. 0·2 per cent nitrogen mustard in acetone.

FIG. 3.—3 days after 2nd weekly application of the same.

FIG. 4.—3 days after application of 0·3 ml. 2 per cent " R48".

FIG. 5.—3 days after 2nd weekly application of the same.

FIG. 6.—3 days after 2nd weekly application of 0·9 ml. 20 per cent urethane in acetone.

FIG. 7.—6 days after 18th weekly application of 0·3 ml. 20 per cent urethane in acetone.

FIG. 8.—3 days after application of 0·2 ml. 0·15 per cent 9 : 10-dimethyl-1 : 2-benzanthracene in acetone.

Note that the epidermal hyperplasia in skin treated with NM, R.48, and DMBA is of the same general type. Note also the absence of hyperplasia in skin treated with urethane. For further description of these appearances see text.

Salaman and Roe.

6

7

8

Salaman and Roe.

in 14 surviving mice after 20 weeks' treatment with croton oil, which suggested that NM had some initiating power ; but the number of mice was too small for a definite conclusion to be drawn. A further test of this substance is planned.

It might be argued from the results of the second experiment that urethane is a much weaker initiator than are carcinogens such as DMBA, since the dose of the former was about 1,000 times that of the latter for comparable effect. Such reasoning would not be justified. The dose of urethane given may have been more than the optimum (cf. Shubik and Ritchie, 1953), and a smaller dose might have proved equally or even more effective. This possibility is being investigated. It is probable, however, that urethane, a very soluble and rapidly eliminated drug, would need to be applied to the skin in much higher concentration, for comparable effect, than the carcinogenic hydrocarbons which persist in the skin for several days at least. In work of this kind the concentration of applied substances is not by itself of much significance. The dose which reaches the site of action in the tissue depends on many factors, of which the applied concentration is only one.

The choice of concentrations was actually based on several considerations. The applied doses of R48 and NM were such as to produce slight but definite epidermal hyperplasia—presumptive evidence that they had reached the susceptible cells. Urethane was used in doses which caused transient narcosis, showing that it had at least traversed the skin. Solubility in acetone was a limiting factor in the cases of BY and AAF, but the doses given were considerably in excess of those previously given by other routes. It is possible that the use of larger doses might have revealed activity in some of the substances which appeared inactive, but in the present work the primary object was to select substances with easily demonstrable initiating action. The detection of minor degrees of activity has been deferred.

The histological appearances of skin after treatment with the various substances were of considerable interest. AAF produced no detectable abnormality and BY only a very slight epidermal hyperplasia, after 2 weekly applications. NM and R48, however, produced after 3 and 10 days a type of epidermal hyperplasia (Fig. 2, 3, 4, and 5) chiefly characterised by gross irregularity in size and arrangement of cells, which, as Pullinger (1940) has shown, is typical of the effects of carcinogenic as opposed to non-carcinogenic irritants. For comparison a section of mouse skin 3 days after a single application of 0·15 per cent DMBA in acetone is shown (Fig. 8). Even after 18 weekly applications of urethane both naked-eye and microscopic appearances of the skin were normal (Fig. 6). Thus we have the apparently anomalous result that two substances, in doses which produced reactions in the skin typical of the early effects of carcinogens, were inactive (or, in one case, perhaps slightly active) as initiators, while another substance which gave no detectable skin reaction was an effective initiator.

The mice treated with urethane and croton oil have been under observation for 7 months. So far the tumours which have arisen on the treated skin are all benign in appearance. Only benign tumours are produced by a small dose of a carcinogen followed by croton oil during the first 6 months or more (Shubik, 1950). As mentioned above, experiments recently completed in this laboratory show that in mouse skin given the latter type of treatment malignant tumours appear at a later date, many of them without obvious relation to pre-existing benign tumours. It is quite possible that malignant tumours will still appear in the mice treated with urethane and croton oil, which are still under observation.* There is as yet no evidence to distinguish urethane and DMBA with respect to the power of initiating the development of malignant tumours.

SUMMARY.

1. The effects on the dorsal skin of mice of various doses of 2-acetylamino-fluorene (AAF), p-dimethylaminoazobenzene (butter yellow, BY), ethyl carbamate (urethane), methyl bis (β-chloroethyl) amine hydrochloride (nitrogen mustard, NM), and NN-di-(2-chloroethyl)-β-naphthylamine (R48), followed by weekly applications of 0·5 per cent croton oil in acetone, were studied.

2. Tumour incidence in the urethane group was significantly greater than in control groups treated with croton oil alone. A few tumours which appeared in the AAF, BY, and R48 groups were probably due to croton oil. NM gave an intermediate result of doubtful significance.

3. In a second experiment the effect on mouse skin of urethane, (*a*) alone, (*b*) following treatment with a carcinogen, and (*c*) followed or accompanied by treatment with croton oil were studied.

4. Weekly doses of 120 mg. urethane followed by 18 weekly applications of croton oil produced large numbers of tumours.

5. Alternate applications of 60 mg. urethane and 0·5 per cent croton oil at 3–4 days' intervals had a similar effect.

6. Urethane alone, either as 2 weekly applications of 120 mg. or as 18 weekly applications of 60 mg., produced no tumours.

7. 18 weekly applications of 60 mg. urethane following an initial application of 0·3 mg. 9 : 10-dimethyl -1: 2-benzanthracene (DMBA) produced no tumours.

8. Urethane produced no recognisable histological changes in mouse skin, even after prolonged application.

9. Tumours produced by urethane in conjunction with croton oil had the appearance of benign papillomata. It is pointed out that, by analogy with the effect of DMBA followed by croton oil, malignant tumours are not to be expected till a later date.*

10. The relation between the effective initiating doses of DMBA and urethane respectively is discussed.

11. It is concluded that urethane is an initiator of carcinogenesis, but not a carcinogen or a co-carcinogen, for mouse skin.

*Addendum.*—After this work was completed our attention was drawn to a recent report by Graafi, Vlamynch, Hoffman and Schulz (1953). The authors have tested the effects on mouse skin of various substances applied alternately with croton oil for periods up to 12 months. 1 : 2-Benzanthracene, and urethane, applied in this way, produced a significantly greater number of tumours than croton oil alone. They were much fewer in number, and occurred a good deal later, than those in our urethane groups, reaching a maximum of 18 in 9 surviving mice in the 1 : 2-benzanthracene group and 12 in 19 surviving mice in the

* A mouse of Group 13, which had received alternate urethane and croton oil applications for 18 weeks, developed a malignant tumour about the middle of the treated area, 8 weeks after the end of treatment. Microscopically, this was seen to be a squamous epithelioma penetrating the panniculus carnosus, and there was a metastasis in the left axillary gland.

urethane group at 12 months. One malignant tumour occurred in their urethane group 6 months after the end of treatment. Pyrene, phenanthrene, acenaphthene, fluorene, aminofluorene, acetylaminofluorene, butter yellow, 3′ methyl butter yellow, and camphor, produced either no tumours, or so few that they might have been due to the croton oil.

With respect to the substances also tested by us (urethane, acetylaminofluorene, and butter yellow) our results confirm theirs.

We are indebted to Miss E. Firth, B.Sc., for skilled technical assistance.

The expenses of this research were partly defrayed out of a Block Grant from the British Empire Cancer Campaign.

### REFERENCES

Berenblum, I.—(1941*a*) *Cancer Res.*, **1**, 44.—(1941*b*) *Ibid.*, **1**, 807.
*Idem* and Shubik, P.—(1947*a*) *Brit. J. Cancer*, **1**, 379.—(1947*b*) *Ibid.*, **1**, 383.—(1949) *Ibid.*, **3**, 384.
Friedewald, W. F., and Rous, P.—(1944) *J. exp. Med.*, **80**, 101.
Furth, J.—(1953) *Cancer Res.*, **13**, 477.—See also Annotation (1953) *Lancet*, ii, 767.
Graffi, A., Vlamynch, E., Hoffman, F., and Schultz, I.—(1953) *Arch. Geschwulstforsch.*, **5**, 110.
Gwynn, R. H., and Salaman, M. H.—(1953) *Brit J. Cancer*, **7**, 482.
MacKenzie, I., and Rous, P.—(1941) *J. exp. Med.*, **73**, 391.
Nettleship, A., and Henshaw, P. S.—(1943) *J. nat. Cancer Inst.*, **4**, 309.
Pullinger, B. D.—(1940) *J. Path. Bact.*, **50**, 463.
Rous, P., and Kidd, J. G.—(1941) *J. exp. Med.*, **73**, 365.
Salaman, M. H., and Gwynn, R. H.—(1951) *Brit. J. Cancer*, **5**, 252.
Shubik, P.—(1950) *Cancer Res.*, **10**, 713.
*Idem* and Ritchie, A. C.—(1953) *Ibid.*, **13**, 343.
Thomson, W.—(1930*a*) *J. Hyg.*, **36**, 24.—(1930*b*) *Ibid*, **36**, 156.